# The Comparison between Effect of Chloralhydrate and Diphenhydramine on Sedating for Electroencephalography

**Published:** 2016

**Authors:** Afagh HASSANZADEH RAD, Vahid AMINZADEH

**Affiliations:** 1 Growth Disorders Research Center, School of Medicine, 17th Shahrivar Hospital, Guilan University of Medical Sciences, Rasht, Iran.

**Keywords:** Conscious Sedation, Diphenhydramine, Chloral Hydrate, Electroencephalography

## Abstract

**Objective:**

Electroencephalography (EEG) is the most effective diagnostic tool in distinguishing epileptic seizure. Chloral hydrate (CH) is a sedative hypnotic drug, commonly used as a method of sedation in children aged<3 yr.

Furthermore, diphenhydramine (DH) is a first generation antihistaminic drug (H1 receptor blocker) with anti-cholinergic effect. In this study, we aimed to compare the effects of CH and DH on sedating for EEG.

**Materials & Methods:**

This retrospective cohort study was conducted on patients’ records of aged 15-72 months undergone an EEG and required sedation. Overall, 200 children were assessed including 100 patients in group 1 (CH) and 100 patients in group 2 (DH). Data were gathered by a form including age, sex, the cause of EEG, complication, success rate, first dose success, as well as sleep and awake latency.

Data were reported by descriptive statistics (mean, standard deviation, number, and percent) and analyzed by t-test and chi-square using SPSS 19.

**Results:**

Totally, 113(56%) male patients with the mean age of 35.62±14.00 months participated in this study. Vomiting and agitation were the most frequent complications in CH and DH groups, respectively. Most of patients in both group indicated successful sedation. CH indicated higher rate of success by first dose toward DH. In addition, CH mentioned lower sleep latency and significant difference was noted between groups. The mean duration of awake latency was higher in DH groups which showed significant difference.

**Conclusion:**

CH might be a more effective drug in comparison with the DH for sedation. According to the availability and low cost of DH, investigators are advised to perform further investigations.

## Introduction

Electroencephalography( EEG) is the most effective diagnostic tool in distinguishing epileptic seizure from other non- epileptic seizures and classifying epilepsy (1).

Performing an appropriate EEG requires children cooperation. Although, EEG following natural sleep can be more valuable but sedation is an appropriate choice when children do not assist (2). So far, various methods have been represented to sedate children. Clinicians prefer to use an easily reversible method with fast efficacy, few complications and adverse effects on cardio respiratory system (3).

Chloral hydrate (CH) is a sedative hypnotic drug commonly used as a method of sedation in children aged <3 yr. It has high gastrointestinal absorption and metabolized to trichloroethanol and trichloroacetic acid in liver, erythrocytes, and other tissues rapidly. 

It can enter in central nervous system and induce sleep by a relatively fast speed and can be urinary excreted. However, it may cause abdominal distension, vertigo, ataxia, headache, paradoxical agitation, cancer (consequent to long-term use), hallucination, nightmare, drug sensitivity, seizure, neonatal jaundice, vomiting, low blood pressure, bad taste and cardiac arrhythmia. 

Trichloroethanol and trichloroacetic acid have 7-11 days and multiple days half-life, respectively (4). It may have small adverse effects on EEG, and can effect fast and be noted as a safe treatment by administering appropriate dosage. Usually, administering 50-75 mg/kg oral CH can sedate children during 15-60 min which lasts 4-9 h (5).

Furthermore, diphenhydramine (DH) is a first generation antihistaminic drug (H1 receptor blocker) with anticholinergic effect. It is commonly used for treating allergy, movement disorder, and extraparamidal side effects. It has sedative hypnotic effects. Although, it is a proper method with less threatening for respiratory depression but it can induce paradoxical agitation and low blood pressure, vertigo, vomiting, nausea, arrhythmia, dry mouth, urinary retention, blurring, and tremor. The proper dose of DH for sedation is 1.2 mg/ kg. The sedative effect will be initiated during 30-60 min and will last 4-7 h (6, 7).

In this study, we aimed to compare the effects of CH and DH on sedating for EEG.

## Materials & Methods

This retrospective cohort study was conducted on patients’ records referred to 17 Shahrivar Hospital, Rasht, Iran during 2009- 2012. The patients aged 15-72 months undergone an EEG and required sedation. Their chief complaint were first unprovoked seizure (no drug consumption), or seizure mimicking events, or headache (no drug use), febrile convulsion (after 2 weeks), and/ or speech disorders without developmental defects. 

Children with drugs allergy, liver or kidney dysfunction, severe cardiac diseases, peptic ulcer, severe systemic diseases, life threatening diseases, history of sedative hypnotic drug use during preceding 48 h, and current systemic diseases were excluded. Overall, 200 children were assessed including 100 patients in group 1 (CH) and 100 patients in group 2 (DH). 

CH with dose of 25 mg/ kg was administered for group 1. No response indicated repeated dose for each 30-60 min up to 120 mg/kg (for infants) and 2 gr (for children) (8). For group 2, 1.2 mg/kg DH was administered and consequent to no response, half dose of DH was administered.

The depth of sedation was assessed by 5 points University of Michigan sedation scale (9). Patients with≥ stage 1 of sedation underwent an EEG. The EEG was performed by Analogus machine, 21 scalp position and international 10-20 system after successful sedation. Trained personnel assessed patients after EEG and recorded the results completely. Data were gathered by a form including age, sex, the cause of EEG, complication, success rate, first dose success, as well as sleep and wake latency.

Vital signs were measured before enrollment and each 15 min up to 2 h (at discharge) after EEG. Lack of sedation or the presence of complication indicated unsuccessful method of sedation.

Data were reported by descriptive statistics (mean, standard deviation, number, and percent) and analyzed by t-test and chi-square using SPSS 19 (Chicago, IL, USA).

## Results

Overall, 200 records of patients with the mean age of 35.62±14.00 months were assessed in this study. Vomiting and agitation were the most frequent complications in CH and DH groups, respectively (P=0.602). Febrile convulsion and probable seizure were the most frequent causes of EEG in CH and DH groups, respectively.

Most of patients in both group indicated successful sedation, but significant difference was noted between groups (P=0.001).

CH indicated higher rate of success by first dose toward DH (P=0.022). In addition, CH mentioned lower sleep latency and significant difference was noted between groups (P=0.001). The mean duration of wake latency was higher in DH groups which showed significant difference (P=0.001) (Table 1).

**Table 1:** Comparing Demographic and Therapeutic Characteristics between CH and DH Groups.

	**CH group**	**DH group**	***P*** **-value**
**Age ( mean±SD)**	34.15±14.09	37.09±13.8	0.138*
**Sex Number (%)**			
**Female** **Male**	45 42	55 58	0.776**
**Successful sedation Number (%)**			
**Yes** **No**	97 3	76 24	0.001**
**First dose sedation Number (%)**			
**Yes** **No**	90 7	63 15	0.022**
**Sleep latency( mean±SD)**	23.45±7.5	33.06±7.36	0.001*
**Awake latency( mean±SD)**	41.97±12.5	62.8±14.4	0.001*
**Complications Number (%)**			
**Yes** **No**	9 91	6 83	0.602**
**Types of complication Number (%)**			
**Agitation** **Ataxia** **Tachycardia** **Vertigo** **Vomiting**	1 1 0 1 7	5 0 1 0 0	0.013**
**Causes of EEG Number (%)**			
**FUS** **FC** **Unprovoked epilepsy** **Other**	27 28 24 21	31 9 32 28	0.007**

* independent T test

** chi-square

## Discussion

Investigators compared CH and DH in patients requiring EEG. As DH is an easy administrable, cheap and accessible drug and CH is a synthetic drug, which may be inaccessible and as there is no previous investigation, to compare these drugs, we assessed them concomitantly in this study. 

Results showed that most of the patients in both groups had successful sedation but CH showed higher success rate. This result was consistent with Fallah et al., which reported 96.7% success rate by administering CH and 70% success by Promethazine, an antihystamine drugs (10). In addition, bektas et al. assessed patients sedated by CH and hydroxyzine (an anti-histamine drug) and noted that 89% of patients with CH and 89.6% of patients with hydroxysine successfully sedated (11). Roch et al. noted consistent results for sedating children undergoing echocardiogaraphy. They demonstrated chloral hydrate as the fast agent with a high success rate (12). But, Favero et al. noted lower success rate by administering CH for sedation induction (56%) (13). 

We found lower sleep latency by CH consistent with Fallah et al. They noted shorter sleep and wake latency by CH in comparison with promethazin (10). The mean sleep latency by CH and hydroxysine were respectively 32.3±26.8 min and 34.68±30.75(11). Ashrafi et al. assessed success rate of sedation by chloral hydrate and melatonin and noted similar results for sleep onset latency in groups, but longer sleep latency and drowsiness were noted by CH compared to melatonin (14). 

Fallah et al noted. no significant difference between groups regarding complication and indicated CH as a safe and effective method, also they mentioned vomiting in CH group (20%) higher than our results (10). Bektas et al. noted more prominent side effects of CH compared to hydroxysine in sedating for EEG (11). In addition, Heistein et al. revealed serious complications including apnea, airway obstruction, hypoxia, hypercarbia and hypotension by administering CH (15).

Previous investigation which assessed oral sedation with midazolam and DH or midazolam in children undergoing magnetic resonance imaging revealed that combination of midazolam and DH noted less sedation failure and can be more advantageous than midazolam alone (16).

**In conclusion, **CH can be a more effective drug in comparison with the DH for sedation. According to the availability and low cost of DH, investigators recommend performing further investigations on both drugs. 
